# Prenatal and clinical characteristics of pregnant women infected with COVID-19 in Yazd, Iran: A multicenter cross-sectional study

**DOI:** 10.18502/ijrm.v20i7.11555

**Published:** 2022-08-08

**Authors:** Razieh Sadat Tabatabai, Leila Asadi, Maryam Mohammadi, Mina Rahmani, Elahe Rezaeian, Fatemeh Ghasemi, Mohammad Javad Tarahi

**Affiliations:** ^1^Department of Gynecology and Obstetrics, Mother and Newborn Health Research Center, Shahid Sadoughi University of Medical Sciences, Yazd, Iran.; ^2^Department of Midwifery, Research Center for Infection Disease, Shahid Sadoughi University of Medical Sciences, Yazd, Iran.; ^3^Department of Midwifery and Reproductive Health, School of Nursing and Midwifery, Isfahan University of Medical Sciences, Isfahan, Iran.; ^4^Department of Midwifery, Research Center for Nursing and Midwifery Care, Shahid Sadoughi University of Medical Sciences, Yazd, Iran.; ^5^School of Medicine, Shahid Sadoughi University of Medical Sciences, Yazd, Iran.; ^6^Shahid Sadoughi University of Medical Sciences, Yazd, Iran.; ^7^Departments of Epidemiology and Biostatistics, Isfahan University of Medical Sciences, Isfahan, Iran.

**Keywords:** Coronavirus, COVID-19, Pregnancy, Clinical, Prenatal.

## Abstract

**Background:**

Coronavirus infection has caused widespread concern among mothers and physicians about the health of pregnant women and infants.

**Objective:**

The aim of this study was to evaluate the clinical and prenatal findings of pregnant women with coronavirus disease-2019 (COVID-19) virus.

**Materials and Methods:**

The present study was a descriptive study that was conducted in 6 mother and child care centers. In this study, 81 pregnant women with COVID-19 admitted to centers in the period from March 2020-September 2020 were studied. Clinical and prenatal findings of the pregnant mothers were recorded using a data collection form with details of demographic characteristics and these were analyzed.

**Results:**

The gestational age of the affected women was between 4 and 40 wk. 48 deliveries were performed and 25% of deliveries were preterm. Coronavirus infection was the cause of termination of pregnancy in 4 cases. The most common symptoms of women when visiting the medical centers were: dry cough (58.0%), muscle pain and myalgia (56.8%) and fever (51.9%). The most common laboratory findings in the women were: increased C-reactive protein (67.90%), lymphopenia (18.51%), decreased white blood cells (27.16%), and increased liver enzymes (18.51%). Regarding the status of the newborns, out of the 33 neonates examined, 3 neonates were diagnosed with COVID-19.

**Conclusion:**

The most common symptoms of pregnant women with COVID-19 are similar to those of other adults. In relation to neonatal infection, given that a number of the neonates tested positive, there appears to be evidence of vertical transmission, which requires further investigation.

## 1. Introduction

Coronavirus disease-2019 (COVID-19) is a disease that has been associated with a rapid increase in morbidity and mortality since its first diagnosis in Wuhan, China in December 2019. The outbreak of COVID-19 became a major epidemic in China from February 2020 (1) and then spread rapidly worldwide. In January 2020, the World Health Organization issued a statement identifying the new coronavirus as the 6 ${}^{\text{th}}$ leading cause of public health emergencies worldwide and identifying the new coronavirus as a threat to all countries (2 3). The World Health Organization's International Classification Committee has identified the causative agent of COVID-19 as the SARS-cov-2 virus (4). The China National Health Commission published a set of guidelines for the prevention, diagnosis, and treatment of COVID-19 pneumonia, as well as epidemiological and clinical features (5). Following the outbreak of the coronavirus crisis worldwide, the outbreak of the virus in Iran was officially confirmed by health officials in February 2020 (6), although cases of suspected COVID-19 disease had been previously reported (7, 8).

The spread of COVID-19 is mainly caused by respiratory droplets transmitted from the infected person by coughing or sneezing, similar to influenza or other respiratory conditions. There are a wide range of symptoms, from general to severe respiratory illness and death (9). COVID-19 can be transmitted to others even from a carrier without symptoms (10). According to studies, the average incubation period in this disease is 5 days (range: 2-14 days) and its symptoms include fever, cough, myalgia, headache, diarrhea and gastrointestinal symptoms. Also, according to the findings, acute respiratory distress syndrome occurs in 27-29% of hospitalized patients. In general, the mortality rate of this disease seems to be 1%. However, based on new data there may be higher mortality rates (11).

Limited information is available on the incidence of COVID-19 during pregnancy. However, information on other diseases associated with highly pathogenic coronaviruses such as SARS and Middle East respiratory syndrome can be useful in raising awareness of the effects of COVID-19 during pregnancy. Various studies have shown that emerging infections can have a significant impact on the health of pregnant women and their fetuses (12, 13). For example, in 2009 some maternal and fetal complications increased with the outbreak of the H1N1 influenza virus (14, 15). In times of rapidly increasing incidence of disease that could have a significant impact on the general health of the community and the medical infrastructure, the needs of pregnant women should be included as a matter of urgency in preparedness and response programs. In similar outbreaks in the past, we have seen that physicians are sometimes reluctant to treat or vaccinate pregnant women due to concerns about fetal safety (16). In establishing monitoring systems for COVID-19 cases, it is essential to collect and report information on clinical findings in pregnancy as well as maternal and fetal outcomes (11).

Therefore, the aim of this study was to evaluate the clinical and prenatal findings of pregnant women with COVID-19.

## 2. Materials and Methods

In this cross-sectional study, all women with COVID-19 referred to 6 mother and child care centers in Yazd city, Iran were studied. The study population consisted of women who were registered on the national system of the Medical Care Monitoring Center for high-risk pregnant women from the beginning of March 2020 (the beginning of the virus epidemic in Iran) until the end of September 2020. Data were collected using a demographic questionnaire and a researcher-made data collection checklist in accordance with the research variables (which were based on previous studies and adjusted according to the research objectives).

All recorded data of pregnant women with COVID-19 at the Medical Care Monitoring Center for high-risk pregnant women were collected during the mentioned period. The participant's clinical condition was extracted based on their documents. In cases of incomplete information, physicians and caregivers were asked to complete the information.

The inclusion criteria in the present study were: COVID-19 ${}^{+}$ pregnant women of any gestational age who were registered in the medical care monitoring system of high-risk mothers, having symptoms of COVID-19 according to the diagnostic algorithms in pregnancy announced by the Ministry of Health. The exclusion criteria included files that did not have enough data and were unable to be completed.

### Ethical considerations

The present study is the result of a research project approved in the second session of reviewing the research projects participating in the call for obtaining a COVID-19 grant from Shahid Sadoughi University of Medical Sciences, Yazd, Iran with letter number 19239 dated 25/4/2020. It also has an ethics code in the bioethics system (Code: IR.SSU.REC.1399.050).

### Statistical analysis

The research data were categorical and numerical (discrete and continuous). Due to the fact that in this study we examined the cases, the data were not normalized and were measured by rank, nominal and relative scales. Descriptive statistics methods (mean, maximum, minimum, standard deviation, frequency distribution) were used to analyze the data and an error of 0.05 was considered for all tests. The Statistical Package for the Social Sciences (SPSS) version 16 was used (IBM, USA, 2007).

## 3. Results

A total of 81 pregnant women with COVID-19 from 6 mother and child care centers were studied according to the inclusion criteria. Of these, 73 (90.1%) were hospitalized in public hospitals and 8 (9.9%) in private hospitals. 24 participants were first gravid, 21 were second, 18 were third gravid, and 18 participants were ≥ 4 ${}^{\text{th}}$ . The mean age of the pregnant women was 30.04 ± 0.71 yr with a gestational age of 4-40 wk, and in the hospitalized participants, the mean age of the pregnancy was 30 wk (Table I). Based on the results, the average number of hospitalization days in women was 3.73 ± 40.00 days (Table I). Data examination showed that 46 (56.79%) women had a history of contact with a person infected with the virus. Other family members were infected in 35 cases (43.20%). Examination of the medical records of the pregnant women showed that the most commonly reported diseases in the pregnant women were, in order of frequency, hypothyroidism, cardiovascular disease, and gestational diabetes (Table I).

Participants' symptoms were examined at the time of referral and it was found that, in order of frequency, dry cough, myalgia and fever had the highest prevalence of the symptoms (Table II).

The results of the laboratory findings showed that the most reported disorder was related to an increase in C-reactive protein (CRP) (55 cases [67.90%]). Lymphopenia, elevated liver enzymes, and decreased white blood cells were seen in 15 (18.51%), 15 (18.51%), and 22 (27.61%) cases, respectively. The pulmonary findings showed that in 28 (34.56%) cases the blood oxygen saturation level was ≤ 95% and in 53 (65.43%) was > 95%. Of the 16 cases examined by lung CT scan, 13 (81.25%) had pulmonary involvement. 32 (39.50%) women received supportive oxygenation and 12 (14.8%) were referred to the intensive care unit for further services. According to our study, 2 (2.46%) cases used mechanical ventilation and no deaths were reported. The prescribed drugs were categorized into 5 groups: antivirals, antihistamines, nonsteroidal anti-inflammatory drugs, antibiotics, and corticosteroids. In total, the most commonly prescribed drugs were cefazolin, acetaminophen, betamethasone, Kaletra, meropenem, interferon, and azithromycin, in order of frequency (Table III).

The perinatal findings showed that 48 out of the 81 women gave birth during the study period and the rest of the women were discharged after receiving hospital treatment or were still hospitalized at the end of the study period. Out of the 48 deliveries, 29 deliveries were performed by cesarean section and 19 deliveries by normal vaginal delivery, of which 25% were preterm deliveries. Participant management showed that in 34 cases, breastfeeding started immediately after delivery, following approved health protocols and provided training. 31 of the women were discharged from hospital without childbirth and 2 cases were discharged from hospital after spontaneous and missed abortions at wk 17 and 18. The case study showed that in 24 cases the pregnancy was terminated, of which only in 4 cases was the COVID-19 infection the main reason for termination. Other causes of termination were: fetal distress, preeclampsia, miscarriage, placental abruption, intrauterine fetal death, and fetal abnormalities (7, 5, 5, 1, 1, and 1, respectively).

The demographic characteristics of the infants born from the infected mothers showed that the mean age of the infants at birth was 30.95 ± 0.96 wk with a mean neonatal weight of 2.63 ± 30.80 kg. Out of the 48 deliveries, 20 cases were boys and 28 cases were girls. A total of 33 infants were examined for infection using their amniotic fluid and cord blood. 3 cases had COVID-19 and 30 cases were healthy. The neonatal clinical findings showed that 3 neonates had intrauterine growth restriction. Evaluation of the 1 ${}^{\text{st}}$ and 5 ${}^{\text{th}}$ min newborn Apgar scores showed that the 1 ${}^{\text{st}}$ min Apgar score in 2 infants and the 5 ${}^{\text{th}}$ min Apgar score in 1 infant were < 9. In relation to arterial blood gas (ABG) pH, the pH level of 8 infants was ≤ 7.35. Finally, a total of 20 infants needed to be admitted to the neonatal intensive care unit (Table IV).

**Table 1:** Demographic characteristics, medical history and drug use in the pregnant women with COVID-19


**Variable**		**Mean ± standard deviation**
Participant's age (yr)*		30.04 ± 0.71 (19-44)
Gestational age (wk)*		30.95 ± 0.96 (4-40)
Number of hospitalization days*		3.73 ± 3.57 (1-25)
History of other diseases and conditions in pregnancy**		
	**Diabetes mellitus**	2 (2.46)
	**Gestational diabetes**	6 (7.40)
	**Anemia**	2 (2.46)
	**Decreased platelets**	3 (3.70)
	**Hypothyroidism**	14 (17.28)
	**Autoimmune disease**	1 (1.23)
	**Chronic hypertension**	1 (1.23)
	**Preeclampsia**	4 (4.93)
	**Cardiovascular disease**	7 (8.64)
	**Migraine**	2 (2.46)
	**Asthma**	1 (1.23)
Supplements taken during pregnancy**		
	**Iron**	43 (53.08)
	**Multivitamins**	16 (19.75)
	**Folic acid**	19 (23.45)
	**Calcium**	17 (20.98)
	**Omega 3**	3 (3.70)
	**Vitamin D**	7 (8.64)
Other medications taken during pregnancy**		
	**Levothyroxine**	14 (17.28)
	**Types of anticoagulants**	1 (1.23)
	**Metformin**	4 (4.93)
	**Methyl dopa**	2 (2.46)
	**Progesterone**	2 (2.46)
	**Salbutamol**	1 (1.23)
	**Aspirin**	2 (2.46)
*Data presented as Mean ± Standard deviation (Min-Max). **Data presented as n (%)		

**Table 2:** Signs and symptoms of COVID-19 in the pregnant women


**Signs**	**N (%)**
**Fever**	40 (51.9)
**Cough**	47 (58.0)
**Dyspnea**	21 (25.9)
**Chest pain**	6 (7.4)
**Myalgia**	46 (56.8)
**Diarrhea**	7 (8.6)
**Nausea and vomiting**	10 (12.3)
**Olfactory and taste disorders**	10 (12.3)
**Headache**	2 (2.4)
**Earache**	2 (2.4)
**Vertigo**	1 (1.2)
**Sneezing**	2 (2.4)

**Table 3:** Drugs prescribed to the pregnant women with COVID-19


**Medicine**		**N (%)**
**Antiviral**		
	**Kaletra**	18 (22.22)
	**Remdesivir**	7 (8.64)
	**Olstamivir**	1 (1.23)
	**Acyclovir**	1 (1.23)
	**Interferon**	10 (12.34)
**Antihistamine**		
	**Diphenhydramine**	2 (2.46)
**NSAIDs**		
	**Acetaminophen**	43 (53.30)
**Antibiotics**		
	**Hydroxychloroquine**	2 (2.46)
	**Cefazolin**	50 (61.72)
	**Azithromycin**	10 (12.34)
	**Meropenem**	11 (13.58)
**Corticosteroids**		
	**Betamethasone**	24 (29.62)
NSAIDs: Nonsteroidal anti-inflammatory drugs		

**Table 4:** Clinical findings in the neonates born from the pregnant women with COVID-19


		**N (%)**
**COVID-19 infection status**		
	**No COVID-19 infection**	30 (62.50)
	**Infection with COVID-19**	3 (6.25)
	**Was not checked**	15 (31.25)
**Apgar score of the first minute**		
	**≥ 9**	46 (95.83)
	**< 9**	2 (4.16)
**Apgar score of the fifth minute**		
	**≥ 9**	47 (97.01)
	**< 9**	1 (2.08)
**ABG pH**		
	**≤ 7.35**	8 (16.66)
	**7.35-7.45**	40 (83.33)
	**≥ 7.45**	0 (0)
**Hospitalized in the NICU**		
	**Yes**	20 (41.66)
	**No**	28 (58.33)
**Infant mortality and morbidity**		
	**Fever and sepsis**	1 (2.08)
	**Moaning and not breastfeeding**	2 (4.16)
	**Cardiac abnormalities**	1 (2.08)
	**Death**	1 (2.08)
**Weight**		
	**≤ 2500 gr**	13 (27.08)
	**> 2500 gr**	35 (72.91)
ABG: Arterial blood gas, NICU: Newborn intensive care unit		

## 4. Discussion

In the present study, 81 pregnant women with COVID-19 were clinically evaluated from 6 centers. The aim of this study was to evaluate the clinical and prenatal findings of pregnant women with COVID-19. Regarding the clinical findings of the participants, the most common symptoms reported at the time of admission were dry cough, muscle aches, myalgia, fever, sore throat, and dyspnea. In other studies (17-19), the most common symptoms found were fever, cough, shortness of breath, fatigue, and myalgia, which is generally consistent with the results of our research. The results of our study and literature review showed that fever, cough, and myalgia are the most common symptoms of COVID-19 in pregnant women.

According to the laboratory findings, the most commonly reported disorders were increased CRP, lymphopenia, decreased white blood cells, and increased liver enzymes. In previous studies (20-23), an increase in CRP was also reported, which is in line with the results of our research. One study showed that an increase in CRP was seen in 45.7% of cases (24). Also, leukopenia, lymphopenia, increased aspartate aminotransferase and increased creatine kinase were found in another study (25). It seems that the discrepancies in the laboratory findings may be due to the limited number of participants examined in some studies.

Examination of the participants' pulmonary findings showed that in 28 cases, the blood oxygen saturation level was equal to or less than 95%. Of the 16 cases examined by lung CT scan, 13 cases had pulmonary involvement. Moreover, 32 cases needed supportive oxygenation, and 12 were referred to the intensive care unit for additional services. According to our study, 2 cases used mechanical ventilation, and no maternal deaths were reported. A previous meta-analysis showed that the rate of severe pneumonia in the women they examined ranged from 0-14%, and most cases required admission to the intensive care unit. In this study, invasive mechanical ventilation was needed in only a few cases. 2 cases of maternal death were reported in another Iranian study (24).

The most commonly prescribed drugs in the present study were cefazolin, non-steroidal anti-inflammatory drugs (acetaminophen), corticosteroids (betamethasone), and antivirals (Kaletra), in order of frequency. The most prescribed drugs for treating COVID-19 were ribavirin and arubidol. Ceftzol sodium and ceftriaxone were also used to prevent bacterial infections during the cesarean section for pregnant participants. Dexamethasone was used to mature the fetal lung. It should be noted that sodium heparin was used to prevent thrombosis, and diamine glycyrrhizinate was used to protect liver function (25). In the previously mentioned meta-analysis, the drugs prescribed to participants were: 70.7% antibiotics, 37.8% antiviral therapy, 17.6% corticosteroids, and 2 cases of hydroxychloroquine (24). The comparison of prescription drugs in medical centers in our study with other studies shows that most prescribed drugs were similar despite different regional treatment protocols.

In the present study, preterm labor occurred in 25% of cases. In 4 pregnancies, COVID-19 was the main reason for termination of pregnancy. In this regard, most other studies have demonstrated an increase in preterm delivery following COVID-19 (20). In other studies, preterm labor was reported in 6 out of 10 pregnancies (26), and in 6 out of 9 pregnancies (27). It seems that this increase in the number of preterm deliveries could be due to complications from COVID-19. In our study, fetal distress was the second leading cause of termination of pregnancy.

Examination of the participant's management showed that in 34 cases, immediately after delivery, breastfeeding was started in accordance with approved health protocols and provided training. A review of the literature in this regard suggests 2 types of care approaches. In some studies, the mother and infant were quarantined and separated for 14 days, and breastfeeding did not occur (25). In other studies, breastfeeding was performed with strict health recommendations and supervision (28-30).

A total of 33 infants were screened for COVID-19. The examination was performed through the amniotic fluid and cord blood. 3 cases of COVID-19 and 30 healthy cases were reported. In this regard, in many other studies, vertical infection did not occur in any of the infants, and there is no evidence proving that vertical transmission can occur (25, 31). Despite the results of these studies, a meta-analysis showed that out of 163 participants, according to samples taken from amniotic fluid, placenta, and/or umbilical cord blood, 10 were positive for the SARS-CoV-2 virus, 61 newborns were positive for SARS-CoV-2, and 4 out of 92 breastmilk samples showed evidence of SARS-CoV-2. Therefore, according to the results of our study and the meta-analysis, it seems that vertical transfer is not impossible (30).

Examination of the neonatal clinical findings showed that 3 of the newborns had fetal growth restriction. Examination of the neonates' 1 ${}^{\text{st}}$ min and 5 ${}^{\text{th}}$ min Apgar scores showed that 2 infants had a 1 ${}^{\text{st}}$ min Apgar score < 9. Also, the 5 ${}^{\text{th}}$ min Apgar score in 1 infant was < 9. In relation to the ABG pH, in 8 infants the pH level was lower than or equal to 7.35. Finally, a total of 20 infants needed to be admitted to the neonatal intensive care unit. A similar study showed that the Apgar score of the examined newborns in the 1 ${}^{\text{st}}$ min was 8-9 and in the 5 ${}^{\text{th}}$ min was 9-10 (27).

The most important limitation of the present study was the lack of a long follow-up period for pregnant women to evaluate the consequences of the COVID-19 virus. In this regard, the researchers suggest that future studies are conducted to investigate the long-term consequences on motherhood and infancy. Also, the present study was a case study, and testing to assess the incidence of COVID-19 in infants born in the province's hospitals was not mandatory according to national guidelines. As a result, another limitation of the study was that not all infants born to infected mothers were tested for COVID-19. It is therefore suggested that future studies examine the incidence of COVID-19 in infants according to the approved tests. Also, the relationship between infant infection and neonatal complications and consequences should be examined.

## 5. Conclusion

The present study's results showed that the most common symptoms of pregnant women with COVID-19 when visiting medical centers were dry cough, muscle aches, myalgia and fever. These symptoms are similar to those in other adults. The most common laboratory findings in the affected women were increased CRP, lymphopenia, decreased white blood cells, and increased liver enzymes. The most common medical disorders in these women were hypothyroidism, cardiovascular disease, and diabetes. 33 infants were screened for COVID-19, 3 of whom had COVID-19, which may be evidence of vertical transmission.

##  Conflict of Interest

The authors declare that there is no conflict of interest.
